# New insights into tumor size in colorectal cancer: smaller size, higher risk?

**DOI:** 10.3389/fonc.2026.1908104

**Published:** 2026-10-02

**Authors:** Ruipeng Ding, Tianyu Li, Cong Li, Wang Song, Xianhua Gao, Midie Xu, Sheng Wang

**Affiliations:** 1 Department of Colorectal Surgery, Fudan University Shanghai Cancer Center, Shanghai, China; 2 Department of Colorectal Surgery, Changhai Hospital, Naval Medical University, Shanghai, China; 3 Department of Pathology, Fudan University Shanghai Cancer Center, Shanghai, China

**Keywords:** colorectal cancer, machine learning, metastasis, risk factors, tumor size

## Abstract

**Background:**

In colorectal cancer, the AJCC T category is defined by the depth of bowel wall invasion rather than by the absolute size of the primary tumor. The clinical value of tumor size in assessing tumor aggressiveness, metastatic risk, and prognosis remains insufficiently investigated.

**Aim:**

To evaluate the clinical value of tumor size in assessing potential metastatic risk and prognostic stratification in colorectal cancer.

**Methods:**

We retrospectively included 10,513 patients with colorectal cancer who underwent surgical treatment at FUSCC between 2008 and 2020. We integrated tumor size with clinicopathological characteristics to assess metastatic risk using machine-learning models and SHAP-based interpretation. By modeling tumor size as both a categorical and continuous variable, we further investigated its prognostic relevance using Kaplan–Meier survival analysis and Cox proportional hazards regression incorporating restricted cubic splines. Additionally, an independent cohort of 6,739 patients from Changhai Hospital was used for external validation.

**Results:**

Machine-learning identified lymph node metastasis as the primary predictive endpoint, with the CatBoost model achieving the highest AUROC of 0.800. SHAP analysis showed that smaller tumor diameter contributed positively to predicted metastatic risk. Multivariable restricted cubic spline analysis further demonstrated a significant nonlinear association between tumor diameter and OS, with mortality risk increasing as tumor diameter decreased within the approximately 0–3 cm range. Among younger male patients with locally advanced disease, tumors ≤3 cm were associated with significantly worse OS and DFS than tumors >5 cm (both *P* < 0.001). External validation yielded similar findings.

**Conclusion:**

Smaller tumor size may paradoxically indicate higher lymph node metastatic risk and poorer prognosis in a subset of patients with colorectal cancer, particularly younger male patients with locally advanced disease. Tumor size may therefore serve as a complementary factor for postoperative risk stratification beyond conventional TNM staging.

## Introduction

Colorectal cancer (CRC) is one of the most prevalent malignancies worldwide (1). Prognostic assessment and therapeutic decision-making for CRC primarily rely on the TNM staging system proposed by the American Joint Committee on Cancer (AJCC) (2). Unlike breast cancer (3) or lung cancer (4), the T category in CRC is not determined by absolute tumor size but by the depth of tumor invasion through the bowel wall. According to the AJCC 8th edition staging criteria, T_1–2_ denotes tumor invasion of the submucosa and muscularis propria, respectively; T_3_ indicates penetration through the muscularis propria into the subserosa; and T_4_ signifies penetration through the visceral peritoneum. Consequently, the AJCC staging system for CRC emphasizes the depth of local invasion and anatomical extent rather than the size of the primary lesion.

Although CRC tumor size is not incorporated into the existing TNM staging system, the traditional view holds that tumor size reflects, to some extent, the tumor burden, disease progression, and potential biological behavior. For tumors with relatively homogeneous biological behavior, larger primary lesions typically suggest a longer growth duration, higher tumor burden, and may be associated with deeper local invasion, lymph node metastasis, and poorer prognosis. Alese et al. (5), analyzing approximately 61,000 CRC patients from the US National Cancer Database (NCDB), demonstrated that larger primary tumors were associated with more advanced AJCC stage, positive resection margins, and worse overall survival. Feng et al. (6) also found a significant correlation between tumor size and prognosis, suggesting that tumor size may provide additional prognostic information beyond current TNM staging.

However, the clinical significance reflected by tumor size may extend beyond tumor burden or growth duration alone (7). Even within the same TNM stage, anatomical location, and similar histology, tumors can exhibit heterogeneous growth patterns: some tumors primarily display expansive growth, while others exhibit infiltrative growth as the dominant feature (8), potentially demonstrating earlier local invasion or distant dissemination. Integrating previous research findings and clinical observations, we propose that in specific CRC subgroups, a smaller tumor size might represent a biological phenotype characterized by “small size with high invasiveness”. Several studies have observed this size paradox: Huang et al. (9) reported that smaller tumors were associated with worse cancer-specific survival in a cohort of T_4b_N_0–2_M_0_ colon cancer patients, and patients with smaller tumors were more likely to have lymph node metastasis and lymphovascular invasion. Another study (10) further indicated that in T_2a_ colon cancer, a tumor length <3.5 cm or >5 cm was both associated with decreased DFS.

Therefore, based on this background, utilizing the CRC-specific database of Fudan University Shanghai Cancer Center (FUSCC), this study included 10,513 surgically treated CRC patients to systematically analyze the relationship between pathological tumor size and metastatic potential as well as long-term prognosis. We hypothesized that, after controlling for baseline clinicopathological characteristics, a smaller tumor size might indicate a stronger tendency for metastasis and worse oncologic outcomes. This study aims to further elucidate the complex relationship between tumor size and aggressive behavior in CRC and to evaluate the potential value of tumor size as a supplementary risk stratification indicator to the TNM staging system.

## Materials and methods

### Study design

We retrospectively analyzed data from CRC patients who underwent surgical treatment at Fudan University Shanghai Cancer Center between 2008 and 2020. Inclusion criteria comprised: (1) pathologically confirmed CRC diagnosis; (2) availability of key clinicopathological variables including tumor size, tumor location, TNM stage, histologic type, differentiation grade, lymphovascular invasion (LVI), perineural invasion (PNI), mismatch repair status (MMR), metastasis status, and follow-up outcomes. Exclusion criteria were: (1) missing patient identification data; (2) receipt of neoadjuvant therapy; (3) missing or invalid data for variables required for the corresponding analyses. Ultimately, 10,513 patients were enrolled in the institutional cohort. Tumor size (length) was characterized by the maximal tumor diameter obtained from pathology reports on resected cancer specimens. Regional lymph node metastasis and distant metastasis were evaluated either separately as independent outcomes or jointly as a composite metastatic outcome. Patients were stratified by tumor length for subsequent analyses. The study protocol was approved by the FUSCC Ethics Committee and Changhai Hospital Ethics Committee.

To assess the robustness and generalizability of the findings, an independent external cohort comprising 6,739 patients with CRC from Changhai Hospital was included using the same inclusion and exclusion criteria as the FUSCC cohort. Machine-learning models developed in the FUSCC cohort were directly applied to the Changhai Hospital cohort for external validation. In addition, machine-learning models were also independently trained in the Changhai Hospital cohort, with SHAP analysis used for model interpretation and assessment of the reproducibility of tumor size-related effects.

### Statistical analysis

Continuous variables are presented as mean (± SD) or median (IQR) based on their distribution. Between-group comparisons for continuous variables were performed using the independent samples t-test or Wilcoxon rank-sum test, as appropriate. Categorical variables are presented as number (%), with between-group comparisons conducted using Pearson’s χ² test or Fisher’s exact test. Survival analysis was performed using the Kaplan–Meier method, and differences in overall survival (OS) and disease-free survival (DFS) between groups were assessed using the log-rank test. OS was defined as the time from surgery to death from any cause or the last follow-up. DFS was defined as the time from surgery to tumor recurrence, distant metastasis, death from any cause, or the last follow-up. Cox proportional hazards regression models were used to evaluate the associations between clinicopathological factors and survival outcomes. Results are reported as hazard ratios (HR) with 95% confidence intervals and *P* values. All statistical tests were two-sided, with *P* < 0.05 considered statistically significant. Statistical significance levels are denoted as follows: ns, *P* ≥ 0.05; * *P* < 0.05; ** *P* < 0.01; *** *P* < 0.001. All statistical analyses were primarily conducted using R software and relevant packages.

### Machine learning

This study employed tree-based supervised learning models for predictive analysis, evaluating the performance of multiple machine learning classifiers for binary outcome prediction. Categorical variables were converted into dummy variables via one-hot encoding before model inclusion. Missing values in continuous variables were imputed using the median. Undetected, failed detection, or uninterpretable categorical results were uniformly assigned a missing category or excluded based on variable attributes. To mitigate the influence of extreme values on model training, reasonable range constraints were applied to selected continuous variables.

The entire cohort was randomly partitioned into training and testing sets at a 8:2 ratio using stratified sampling to maintain consistent outcome event proportions between sets. We constructed the following classification models: Decision Tree, Random Forest, ExtraTrees, XGBoost, and CatBoost (11–14). All models were trained on the training set, with performance evaluated exclusively on the testing set. Predictive performance was primarily assessed using the receiver operating characteristic (ROC) curve and area under the curve (AUROC). To enhance model interpretability, SHapley Additive exPlanations (SHAP) values (15) were computed for the final model. These machine learning analyses were implemented using Python packages including scikit-learn, xgboost, catboost, and shap.

## Results

### Baseline characteristics

A total of 10,513 patients were enrolled in this study and stratified into a smaller tumor group (n = 7,397) and a larger tumor group (n = 3,116) based on a cutoff of 5 cm (**Table 1**). No statistically significant difference was observed in age between the two groups (59.62 ± 11.61 vs. 59.55 ± 12.92, *P* = 0.815). Statistically significant differences were found between the groups regardinggender, tumor location, pathological T stage, histological type, differentiation grade, and perineural invasion. Compared to the smaller tumor group, the larger tumor group exhibited a higher proportion of males (62.2% vs. 57.6%, *P* < 0.001), a higher proportion of proximal colon tumors (40.0% vs. 16.8%, *P* < 0.001), and a higher proportion of high-T stage disease (*P* < 0.001). Additionally, the larger tumor group had higher proportions of mucinous adenocarcinoma and signet-ring cell carcinoma histological subtypes, poor differentiation (31.9% vs. 21.5%), dMMR status (21.9% vs. 12.9%) and less perineural invasion (22.0% vs. 26.2%). No statistically significant differences were observed between the two groups in terms of lymphovascular invasion, metastasis, or recurrence rates.

**Table 1:** Baseline demographic and clinicopathological characteristics of patients stratified by tumor length with a 5-cm cutoff.

Variable	Smaller group (N = 7397)	Larger group (N = 3116)	*P* Value
Age (Mean ± SD)	59.62 ± 11.61	59.55 ± 12.92	0.815
Gender			<0.001***
Male	4261 (57.6%)	1939 (62.2%)	
Female	3136 (42.4%)	1177 (37.8%)	
Location			<0.001***
Rectum	4416 (59.7%)	1150 (36.9%)	
Distal	1740 (23.5%)	721 (23.1%)	
Proximal	1241 (16.8%)	1245 (40.0%)	
pTstage			<0.001***
T_1_	393 (5.3%)	10 (0.3%)	
T_2_	1538 (20.8%)	226 (7.3%)	
T_3_	2074 (28.0%)	1130 (36.3%)	
T_4_	3392 (45.9%)	1750 (56.2%)	
Pathology Class			<0.001***
Class I	6903 (93.3%)	2658 (85.3%)	
Class II	421 (5.7%)	397 (12.7%)	
Class III	73 (1.0%)	61 (2.0%)	
Differentiation			<0.001***
Well	88 (1.2%)	29 (0.9%)	
Medium	5717 (77.3%)	2094 (67.2%)	
Poor	1592 (21.5%)	993 (31.9%)	
MMR status			<0.001***
pMMR	6444 (87.1%)	2434 (78.1%)	
dMMR	953 (12.9%)	682 (21.9%)	
LVI	2141 (28.9%)	912 (29.3%)	0.756
PNI	1935 (26.2%)	687 (22.0%)	<0.001***
Metastasis	4279 (57.8%)	1752 (56.2%)	0.130
Recurrence	1962 (26.5%)	785 (25.2%)	0.163

Class I: Adenocarcinoma; Class II Adenocarcinoma with mucinous component; Class III: Adenocarcinoma with signet-ring cell component; MMR: Mismatch repair; LVI: Lymphovascular invasion; PNI: Perineural invasion. ***P < 0.001.

### Crude and standardized metastasis rates

The distribution of metastatic status across different tumor size intervals is presented in **Figure 1**. The overall metastasis rate increased within the smaller tumor-size range, peaked at approximately 3–4 cm, and subsequently slightly declined. A similar pattern was observed for lymph node metastasis, whereas distant metastasis rates were substantially lower and remained approximately 20%–30% across most tumor size intervals.

**Figure 1:**
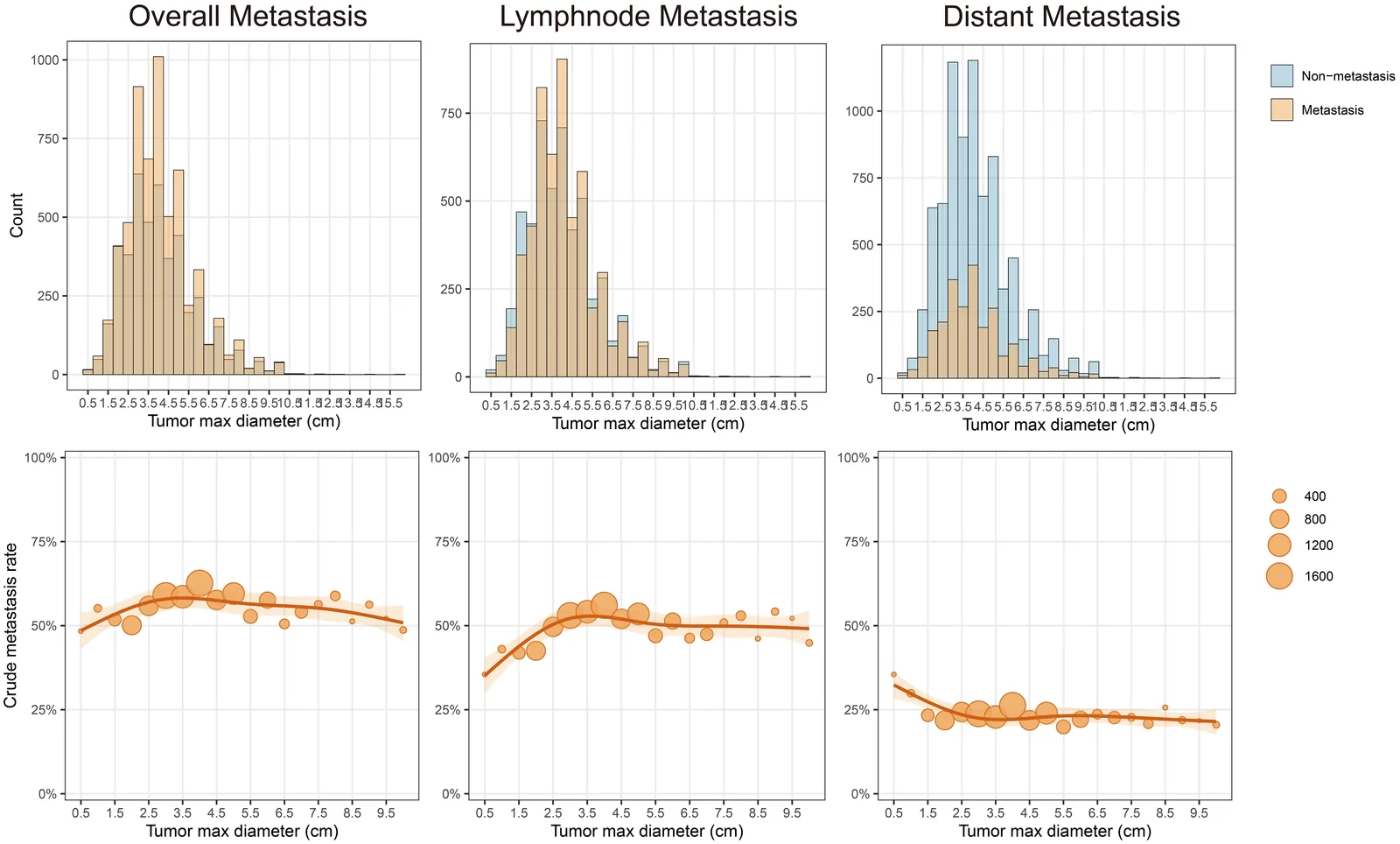
Tumor size-stratified distribution of metastatic status and crude metastasis rates. The upper row shows histograms of non-metastatic and metastatic cases across tumor-size intervals. The lower row shows the crude metastasis rate within each tumor-size interval, with bubble size indicating the number of patients in that interval. From left to right, the columns represent overall metastasis, lymph node metastasis, and distant metastasis.

When grouped using a 5-cm cutoff, the crude overall metastasis rates were 57.8% for the smaller tumor group and 56.2% for the larger tumor group, with no statistically significant difference (*P* = 0.125) (**Figure 2A**). However, after standardization for sex, T stage, tumor location, differentiation grade, and histological type, the adjusted metastasis rate was significantly higher in the smaller tumor group compared to the larger group (59.9% vs. 53.6%, *P* < 0.001) (**Figure 2A**). To evaluate the impact of different tumor length thresholds on metastasis rate comparisons, the tumor length cutoff was incrementally increased from 0.1 cm to 10.0 cm. At each cutoff, patients were re-stratified into smaller and larger tumor groups, and crude metastasis rates as well as adjusted metastasis rates were calculated for both groups (**Figure 2B**). The crude metastasis rates of both groups varied with the changing cutoff and intersected approximately around 4 cm. In contrast, the adjusted metastasis rates demonstrated a more consistent between-group difference across all cutoffs, with the smaller tumor group exhibiting a higher metastasis rate than the larger group at every threshold.

**Figure 2:**
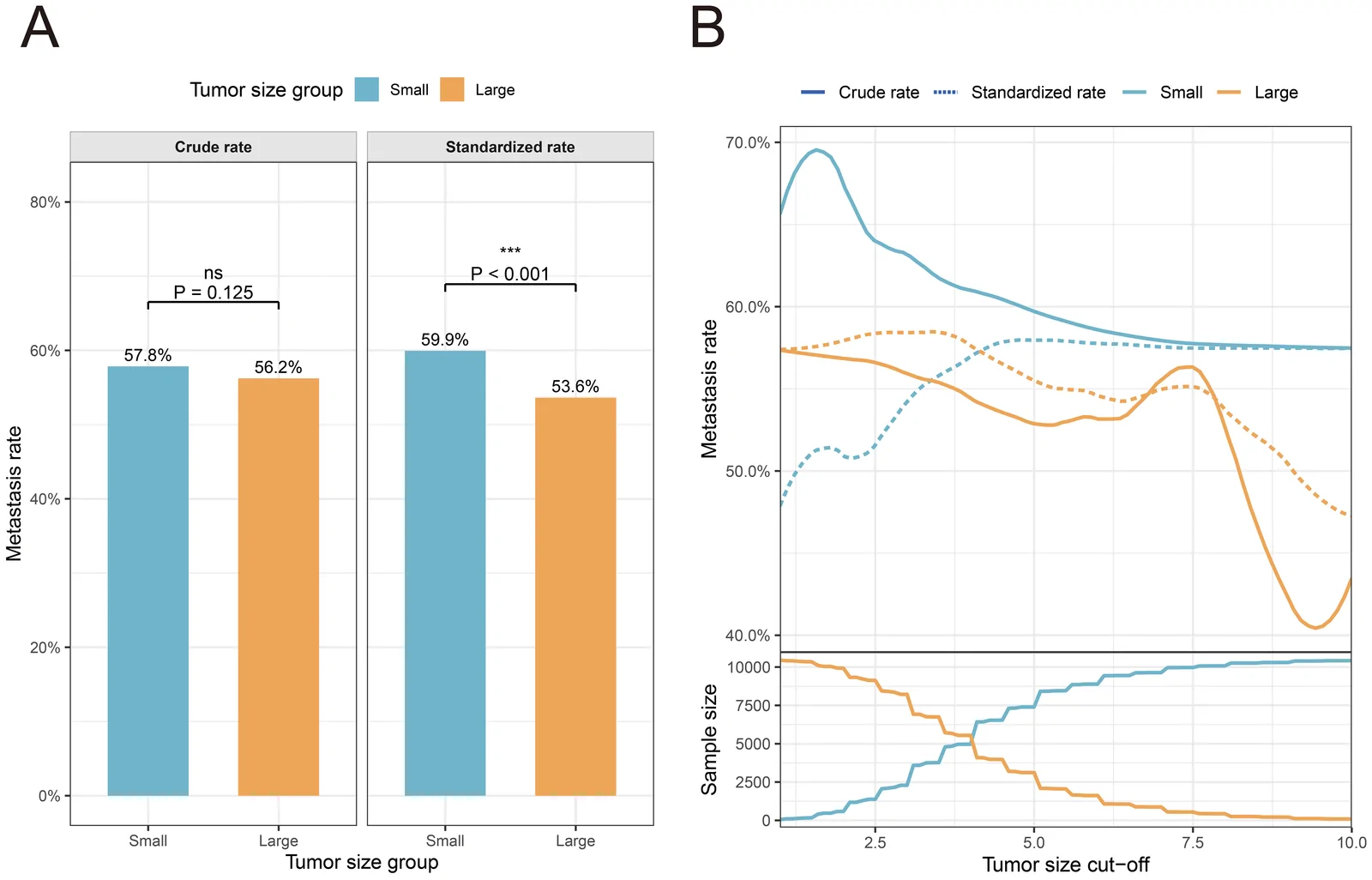
Crude and standardized metastasis rates according to tumor size grouping and serial tumor-size cutoffs. **(A)** Comparison of crude and standardized metastasis rates between small and large tumors defined by a 5-cm cutoff. **(B)** Cutoff-based analysis of crude and standardized metastasis rates across tumor-length thresholds from 0.1 to 10.0 cm, with corresponding group sample sizes shown below. ***P < 0.001.

### Model development and validation

Five tree-based machine-learning algorithms were developed separately for overall metastasis, lymph node metastasis, and distant metastasis (**Supplementary Figure S1**; **Supplementary Tables S1–S3**). For overall metastasis, the AUROCs ranged from 0.767 to 0.779. Model performance was higher for lymph node metastasis, with AUROCs ranging from 0.787 to 0.800, whereas substantially lower discrimination was observed for distant metastasis, with AUROCs ranging from 0.653 to 0.668. Calibration and decision curve analyses also showed more stable performance for overall and lymph node metastasis than for distant metastasis. Therefore, lymph node metastasis was selected as the primary endpoint for subsequent machine-learning analyses.

SHAP analyses showed generally consistent feature contributions across the three metastatic endpoints (**Supplementary Figure S2**). Lymphovascular invasion, pT stage, and perineural invasion were among the major predictive features. Notably, tumor diameter showed a similar pattern across all three endpoints, with smaller tumors generally associated with positive SHAP values and larger tumors with lower or negative SHAP contributions. This pattern remained evident when lymph node and distant metastases were analyzed separately.

For lymph node metastasis, CatBoost achieved the highest AUROC of 0.800 (95% CI, 0.782–0.818), with a Brier score of 0.179 (95% CI, 0.170–0.186) (**Figures 3A–C**; **Supplementary Table S2**). The locked FUSCC models were subsequently applied directly to the independent Changhai Hospital cohort for external validation. External AUROCs ranged from 0.707 to 0.717, with Random Forest achieving the highest AUROC of 0.717, while Brier scores ranged from 0.204 to 0.207. Although discrimination, calibration, and net benefit were attenuated in the external cohort, the models retained moderate predictive performance. For the CatBoost model in the FUSCC cohort, the confusion matrix showed 879 true negatives, 189 false positives, 395 false negatives, and 714 true positives (**Figure 3F**), corresponding to an accuracy of 73.2%, sensitivity of 64.4%, and specificity of 82.3%.

**Figure 3:**
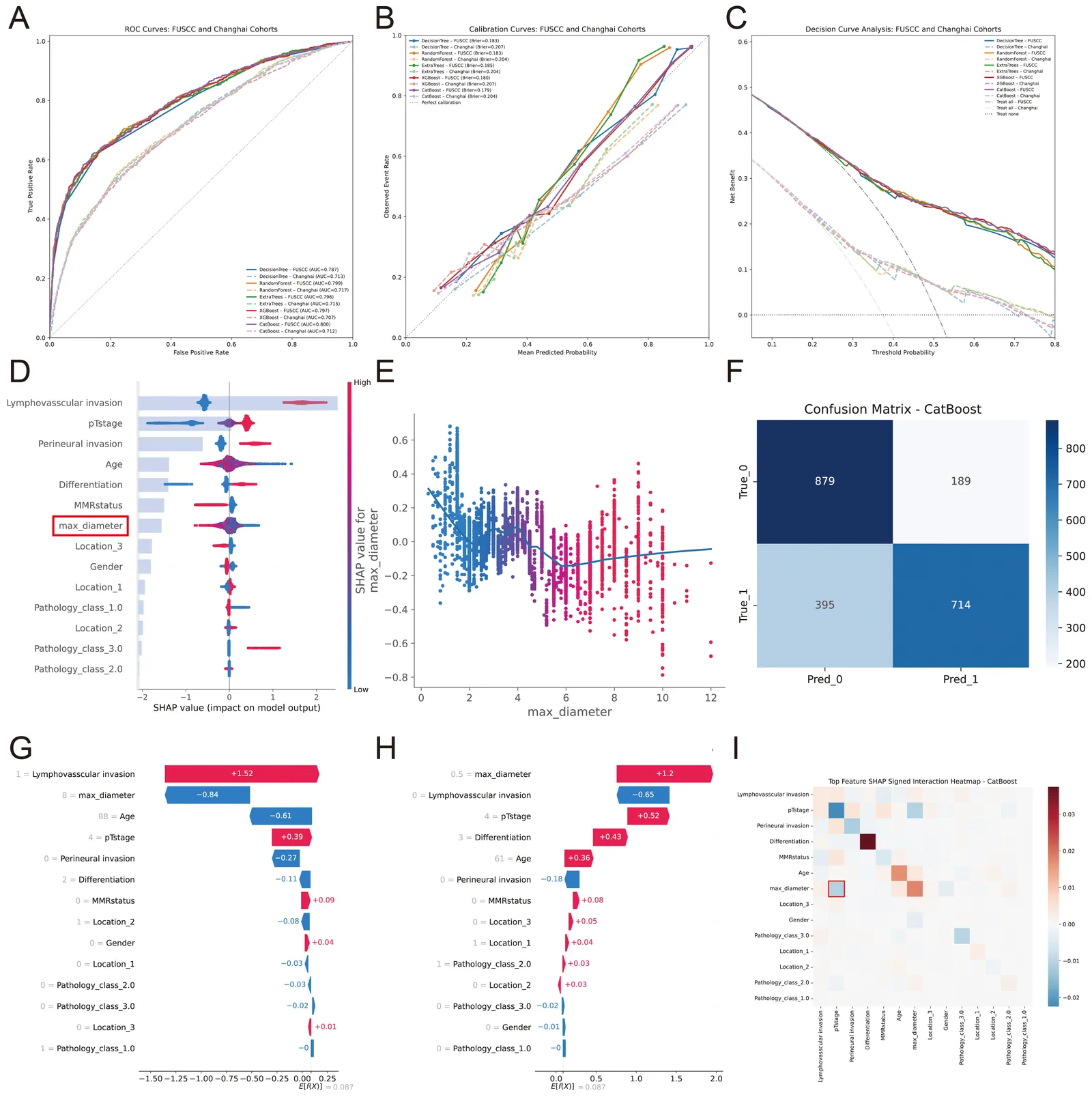
Machine learning model performance and SHAP-based interpretation for lymph node metastasis prediction. **(A-C)**. ROC curves, calibration curves, and decision curve analysis of the five tree-based machine learning models in the FUSCC and Changhai cohorts. **(D)** SHAP summary plot showing the overall feature importance and the distribution of feature-specific SHAP values in the CatBoost model. **(E)** SHAP dependence plot showing the relationship between tumor diameter and its corresponding SHAP value. **(F)** Confusion matrix of the CatBoost model in the prediction set. **(G)** SHAP waterfall plot of the representative sample with the largest positive SHAP contribution from tumor diameter. **(H)** SHAP waterfall plot of the representative sample with the largest negative SHAP contribution from tumor diameter. **(I)** SHAP interaction heatmap of the CatBoost model.

SHAP analysis of the CatBoost model identified lymphovascular invasion as the most influential feature, followed by pT stage, perineural invasion, age, differentiation, MMR status, and tumor diameter (**Figure 3D**). The SHAP dependence plot demonstrated a nonlinear relationship between tumor diameter and lymph node metastasis prediction, with smaller tumors generally contributing positively and larger tumors contributing negatively to the predicted risk (**Figure 3E**). Representative samples with large and small tumor diameters further illustrated strong negative and positive SHAP contributions from tumor diameter, respectively (**Figures 3G,H**). SHAP interaction analysis further revealed a pronounced negative interaction between tumor diameter and pT stage (**Figure 3I**).

To further assess the reproducibility of these findings independently of model transportability, machine-learning models were also retrained within the Changhai Hospital cohort. The SHAP summary plots showed broadly consistent feature patterns between the FUSCC and Changhai cohorts, with lymphovascular invasion, pT stage, and perineural invasion remaining major contributors (**Supplementary Figure S3**).

### Prognostic value of tumor size

Univariable Cox regression analysis showed that increasing tumor length was significantly associated with poorer overall survival (HR = 1.07, 95% CI: 1.05–1.09, P < 0.001). This association remained statistically significant in the multivariable Cox regression model after adjustment for age, sex, tumor location, differentiation grade, histologic type, pT stage, MMR status, lymphovascular invasion, and perineural invasion (HR = 1.04, 95% CI: 1.01–1.06, P = 0.004) (**Table 2**). Although statistically significant, the HR was close to 1.0, reflecting a relatively small effect size.

**Table 2:** Univariable and multivariable Cox regression analyses of clinicopathological factors associated with overall survival.

	Univariable		Multivariable	
Variable	HR (95%CI)	*P* value	HR (95%CI)	*P* value
Tumor Length	1.07 (1.05-1.09)	<0.001***	1.04 (1.01-1.06)	0.004**
Age				
>50	Ref	NA	Ref	NA
≤50	0.78 (0.71-0.86)	<0.001***	0.69 (0.63-0.76)	<0.001***
Gender				
Female	Ref	NA	Ref	NA
Male	1.08 (1.00-1.16)	0.054	1.04 (0.97-1.12)	0.268
Location				
Rectum	Ref	NA	Ref	NA
Distal	0.98 (0.90-1.08)	0.686	0.88 (0.80-0.97)	0.007**
Proximal	1.23 (1.13-1.34)	<0.001***	1.09 (0.99-1.19)	0.075
Differentiation				
Well	Ref	NA	Ref	NA
Medium	1.61 (1.05-2.47)	0.031*	0.98 (0.64-1.51)	0.924
Poor	2.98 (1.93-4.58)	<0.001***	1.36 (0.88-2.11)	0.168
Pathology Class				
Class I	Ref	NA	Ref	NA
Class II	1.11 (0.98-1.26)	0.102	0.94 (0.82-1.08)	0.372
Class III	2.81 (2.21-3.57)	<0.001***	1.46 (1.14-1.87)	0.003**
pTstage				
T1	Ref	NA	Ref	NA
T2	1.60 (1.13-2.27)	0.008**	1.45 (1.02-2.05)	0.040*
T3	3.72 (2.66-5.20)	<0.001***	2.32 (1.65-3.26)	<0.001***
T4	4.42 (3.18-6.15)	<0.001***	3.02 (2.16-4.23)	<0.001***
MMR status				
pMMR	Ref	NA	Ref	NA
dMMR	0.79 (0.71-0.87)	<0.001***	0.76 (0.69-0.84)	<0.001***
LVI	2.45 (2.27-2.63)	<0.001***	1.84 (1.70-1.99)	<0.001***
PNI	2.35 (2.18-2.54)	<0.001***	1.76 (1.63-1.91)	<0.001***

Class I: Adenocarcinoma; Class II Adenocarcinoma with mucinous component; Class III: Adenocarcinoma with signet-ring cell component; MMR: Mismatch repair; LVI: Lymphovascular invasion; PNI: Perineural invasion. **P < 0.01, ***P < 0.001.

In the multivariable model, patients aged ≤50 years had a lower hazard of death than those aged >50 years (HR = 0.69, 95% CI: 0.63–0.76, P < 0.001). Distal colon tumors were associated with a lower mortality risk than rectal tumors (HR = 0.88, 95% CI: 0.80–0.97, P = 0.007), whereas no statistically significant difference was observed for proximal colon tumors (HR = 1.09, 95% CI: 0.99–1.19, P = 0.075). Class III histology was associated with an increased mortality risk compared with Class I histology (HR = 1.46, 95% CI: 1.14–1.87, P = 0.003). The hazard of death increased with advancing pT stage, with HRs of 1.45 for T2, 2.32 for T3, and 3.02 for T4 compared with T1. dMMR status was associated with a lower mortality risk than pMMR status (HR = 0.76, 95% CI: 0.69–0.84, P < 0.001). Both lymphovascular invasion (HR = 1.84, 95% CI: 1.70–1.99, P < 0.001) and perineural invasion (HR = 1.76, 95% CI: 1.63–1.91, P < 0.001) were associated with poorer overall survival (**Table 2**).

Although tumor length showed a statistically significant linear association with overall survival in the multivariable Cox regression model, the HR was close to 1.0, with a relatively small effect size. Further analysis showed a nonlinear association between tumor length and prognosis that was concentrated within specific diameter ranges. Multivariate Restricted cubic spline analysis further showed a significant association between tumor length and overall survival in the multivariable model (P overall = 0.002), with a significant non-linear component (P for non-linearity = 0.036) (**Table 3**, **Figure 4A**). The estimated HR increased with decreasing tumor diameter within the approximately 0–3 cm range. In sex-stratified analyses, the increase in HR with decreasing tumor diameter within the smaller-diameter range was more evident among male patients than among female patients (**Figure 4B**), and In age-stratified analyses, this inverse pattern was more pronounced among patients aged ≤50 years than among those aged >50 years (**Figure 4C**). A nomogram based on the multivariable RCS-Cox model was constructed to estimate 1-year, 3-year, and 5-year overall survival probabilities (**Figure 4D**).

**Table 3:** Multivariable restricted cubic spline Cox regression analysis of clinicopathological factors associated with overall survival.

Variable	N	HR (95%CI)	*P* value	*P_ non-linear*
Tumor Length	10513	NA.	0.002**	0.036*
Age				
>50	8391	Ref	NA	
≤50	2122	0.69 (0.62,0.76)	<0.001***	
Gender				
Female	4313	Ref	NA	
Male	6200	1.04 (0.97,1.12)	0.276	
Location				
Rectum	5566	Ref	NA	
Distal	2461	0.88 (0.80,0.97)	0.007**	
Proximal	2486	1.08 (0.98,1.18)	0.112	
Differentiation				
Well	117	Ref	NA	
Medium	7811	0.99 (0.64,1.52)	0.955	
Poor	2585	1.37 (0.88,2.12)	0.161	
Pathology Class				
Class I	9561	Ref	NA	
Class II	818	0.94 (0.82,1.07)	0.342	
Class III	134	1.48 (1.15,1.90)	0.002**	
pTstage				
T1	403	Ref	NA	
T2	1764	1.55 (1.09,2.22)	0.015*	
T3	3204	2.52 (1.78,3.57)	<0.001***	
T4	5142	3.30 (2.34,4.66)	<0.001***	
MMR status				
pMMR	8878	Ref	NA	
dMMR	1635	0.76 (0.68,0.84)	<0.001***	
LVI	3053	1.84 (1.70,1.99)	<0.001***	
PNI	2622	1.77 (1.63,1.92)	<0.001***	

Class I: Adenocarcinoma; Class II Adenocarcinoma with mucinous component; Class III: Adenocarcinoma with signet-ring cell component; MMR: Mismatch repair; LVI: Lymphovascular invasion; PNI: Perineural invasion. **P < 0.01, ***P < 0.001.

**Figure 4:**
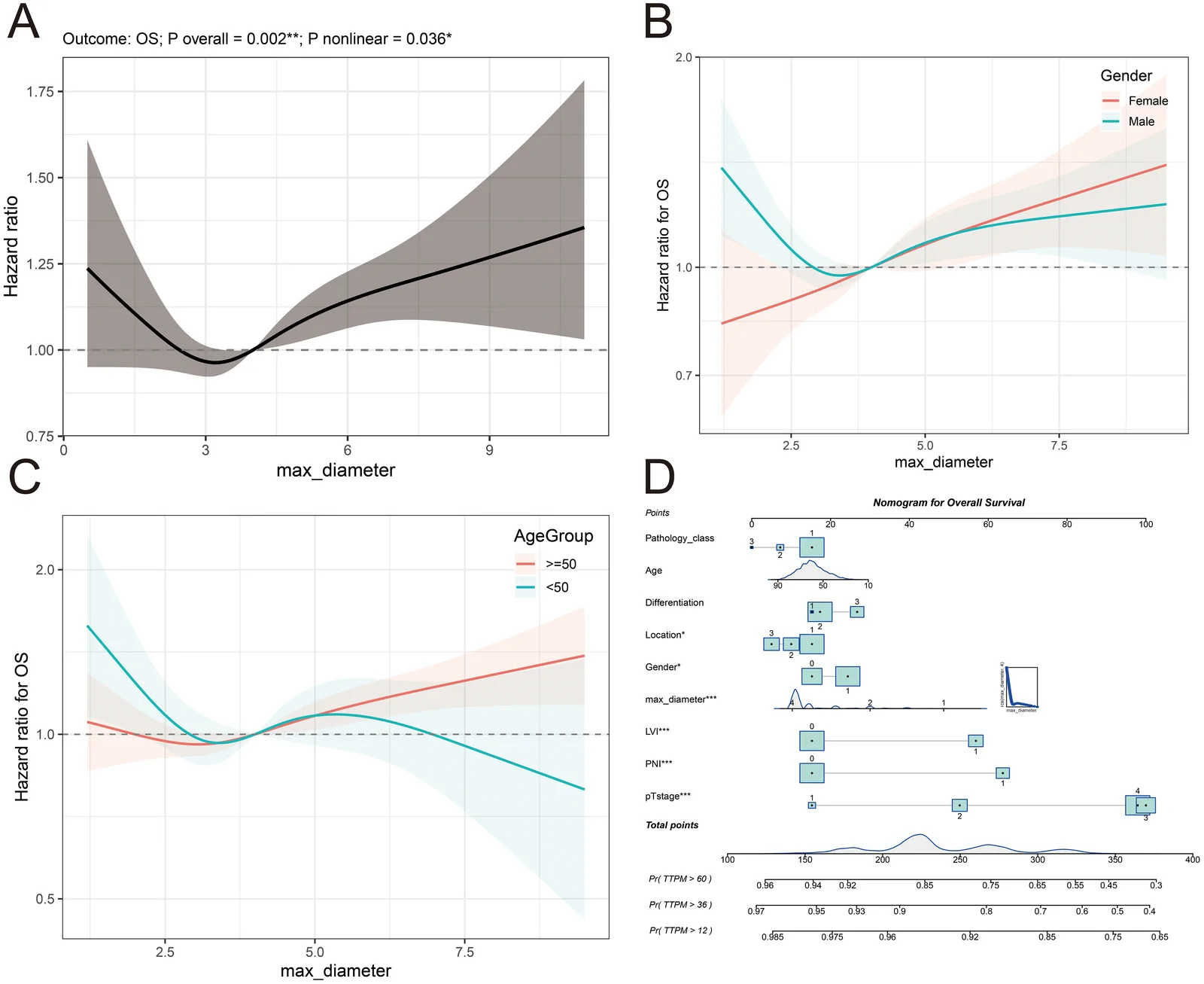
Restricted cubic spline Cox regression analysis of tumor maximum diameter for overall survival. **(A)** Restricted cubic spline Cox curve showing the association between tumor diameter and hazard ratio for overall survival. **(B)** Stratified restricted cubic spline curves according to sex group. **(C)** Stratified restricted cubic spline curves according to age group. **(D)** Nomogram derived from the multivariable restricted cubic spline Cox model for estimating 1-, 3-, and 5-year overall survival probabilities. *P < 0.05, ***P < 0.001.

### Survival analysis

Based on these findings, patients were further stratified into a small-tumor group (≤ 3 cm) and a large-tumor group (> 5 cm). Young (≤ 50 years) male patients with advanced (T4) disease were selected according to the machine learning and Cox regression results. OS and DFS were compared in the overall cohort and in subgroups stratified by metastasis status (**Figure 5**).

**Figure 5:**
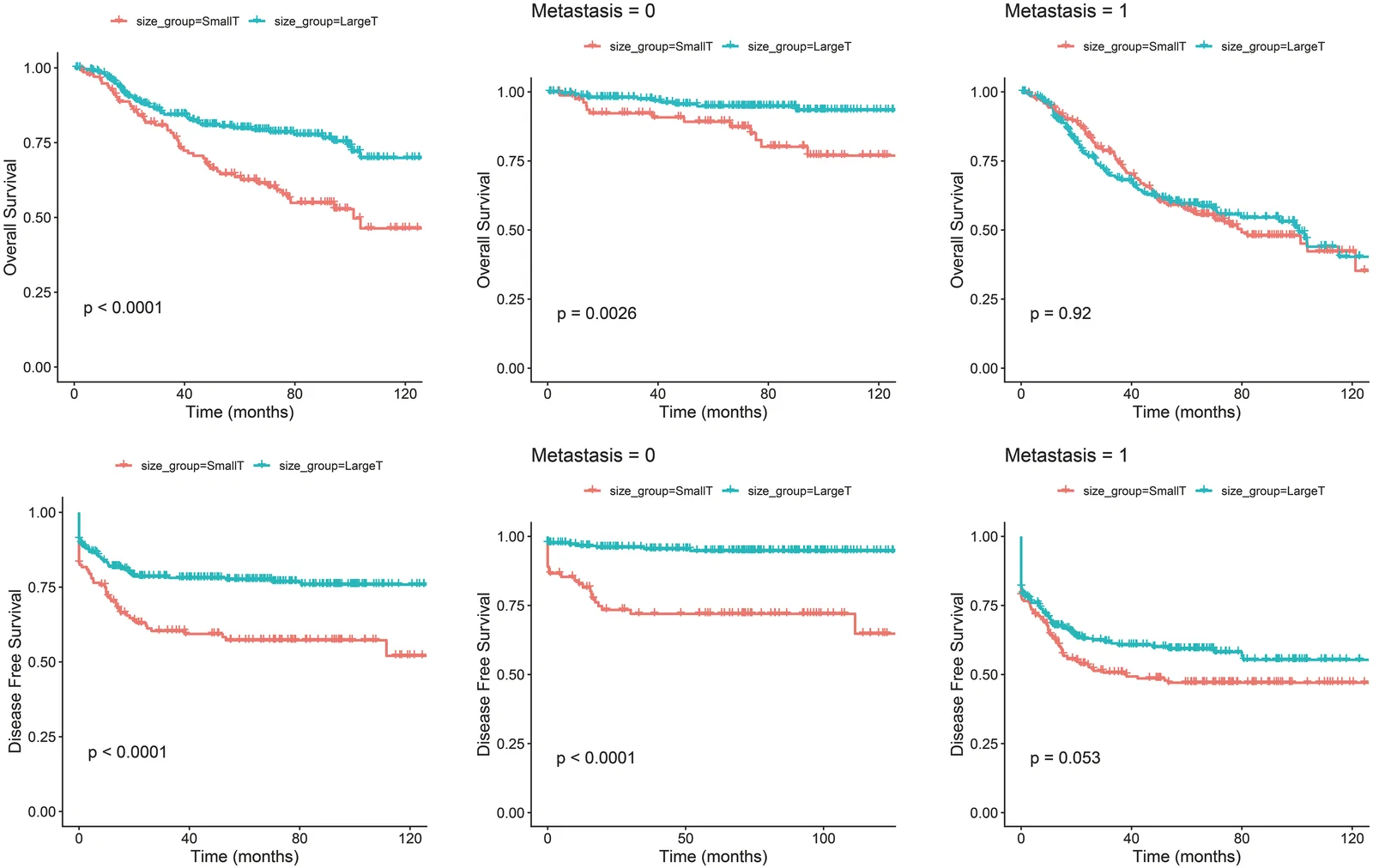
Kaplan–Meier analysis of overall survival and disease-free survival. Patients were divided into small-tumor (≤3 cm) and large-tumor (>5 cm) groups. The upper row shows overall survival, and the lower row shows disease-free survival. Survival curves are shown for the overall cohort and further stratified by metastatic status.

In the overall cohort, the small-tumor group exhibited significantly lower OS than the large-tumor group. The survival curves diverged early during follow-up and maintained this difference thereafter (*P* < 0.001). When stratified by metastasis status, Among patients without metastasis, OS was also significantly lower in the small-tumor group (P = 0.0026). In contrast, no significant difference in OS was observed between the two tumor-size groups among patients with metastasis (P = 0.92).

Similarly, DFS was significantly lower in the small-tumor group in the overall cohort (P < 0.001). Among patients without metastasis, the small-tumor group also had significantly poorer DFS than the large-tumor group (P < 0.001). Among patients with metastasis, DFS was numerically lower in the small-tumor group, although the difference did not reach statistical significance (P = 0.053).

## Discussion

This study systematically evaluated the relationship between pathological tumor diameter and both metastasis risk and long-term prognosis in a large-scale CRC surgical cohort from FUSCC, with additional validation in an independent cohort from Changhai Hospital. Previous research on CRC tumor size predominantly employed 5 cm as an empirical or statistically derived cut-off to investigate its association with recurrence, metastasis, and survival outcomes (5, 16, 17). Building upon this foundation, the present study initially used 5 cm as the primary threshold for preliminary analysis. Subsequently, a systematic evaluation of the non-linear effects of tumor size was conducted using continuous threshold scanning, machine learning models, and Cox regression.

We first observed that when using 5 cm as the cut-off, the difference in crude metastasis rates between the smaller (≤ 5 cm) and larger (> 5 cm) groups was not significant. However, after standardization for sex, T-stage, tumor location, differentiation grade, and histologic type, the smaller tumor group exhibited a higher metastasis rate. To further assess the independent predictive value of tumor length for metastasis risk and its complex interactions with other clinicopathological factors, we therefore further evaluated overall metastasis, lymph node metastasis, and distant metastasis as separate predictive endpoints using five tree-based machine-learning algorithms. Previous studies on CRC tumor size largely relied on fixed thresholds or traditional regression models to evaluate its relationship with recurrence and survival, but rarely systematically analyzed the contribution of tumor length to metastasis risk prediction or its interactions with other variables from this perspective (18, 19). Our results showed that predictive performance was strongest for lymph node metastasis, for which CatBoost achieved an AUROC of 0.800 in the FUSCC test set, whereas substantially lower discrimination was observed for distant metastasis. Importantly, SHAP analyses showed a broadly consistent inverse tumor-size pattern across all three metastatic endpoints: smaller tumor diameters tended to contribute positively to predicted metastatic risk, whereas larger diameters tended to contribute negatively. Since the models showed the best and most stable predictive performance for lymph node metastasis, therefore, lymph node metastasis was then selected as the primary endpoint for subsequent machine-learning modeling and model interpretation. SHAP analysis revealed that lymphovascular invasion, T-stage, perineural invasion, histologic type, age, and tumor length were the primary drivers of model-predicted metastasis risk. LVI, PNI, and T-stage ranked highest, aligning with the established pathological understanding of CRC metastasis (20–22) and indicating that the model primarily captured clinically recognized high-risk factors. Notably, however, both age and tumor length demonstrated counterintuitive trends: younger age contributed positively to metastasis risk, suggesting that early-onset CRC may be associated with more aggressive biological features; Additionally, smaller tumor size corresponded to a higher predicted risk of metastasis. Combined with the aforementioned standardized metastasis rate results, this indicates that tumor size may not solely reflect tumor burden or growth duration but could also be influenced by tumor growth patterns and intrinsic aggressiveness.

Furthermore, we identified a significant interaction between tumor length and T-stage: The discriminative power of tumor diameter for metastasis risk was limited at lower T-stages. However, within the T_3_–T_4_ stage group, smaller diameters were associated with higher predicted risk. We propose the following interpretation: T-stage partially reflects the depth of longitudinal invasion and duration of local progression. For tumors at earlier T-stages, a small diameter could arise from either shorter growth duration or represent a highly aggressive phenotype characterized by prominent longitudinal infiltration and early dissemination; thus, diameter alone is insufficient to distinguish these scenarios. Conversely, when a tumor progresses to T_3_ or T_4_ stage yet maintains a small transverse diameter, this is less likely attributable solely to insufficient growth time. Instead, it may indicate a biological characteristic of “limited lateral expansion but prominent longitudinal infiltration.” Consequently, the significance of tumor length in CRC metastasis risk assessment is demonstrably context-dependent and should be interpreted comprehensively alongside T-stage, LVI/PNI status, histologic type, and age. Particularly in locally advanced patients, small tumor size may denote a more aggressive subtype, warranting further attention in subsequent research and clinical risk stratification.

We further employed Cox regression to systematically evaluate the prognostic value of tumor length. We found that tumor diameter exhibited a weak but significant association with poorer OS in both univariate and multivariate Cox regression. After adjustment for age, sex, tumor location, differentiation grade, histological type, pT stage, MMR status, LVI, and PNI, tumor diameter remained statistically significant, although the effect size was modest (HR = 1.04, 95% CI, 1.01–1.06; *P* = 0.004). This finding suggests that, when tumor diameter is modeled as a single linear term, larger tumors are associated with a modest overall increase in mortality risk. However, the significant nonlinear association identified by multivariable RCS-Cox analysis revealed a more complex relationship that was not adequately represented by the average linear effect. In particular, within the smaller-diameter range of approximately 0–3 cm, mortality risk increased as tumor diameter decreased. This inverse pattern was more evident in male patients and in patients aged ≤50 years. This observation is consistent with the increasing global attention to early-onset colorectal cancer, highlighting the need to further evaluate tumor size-related risk patterns in younger patients (23). Previous studies have also indicated that early-onset CRC is more frequently associated with signet ring cell differentiation, perineural invasion, venous invasion, and higher risks of metastasis or local recurrence (24). Gender differences in CRC are similarly evident across multiple dimensions, including incidence, mortality, tumor location, and survival outcomes (25).

Based on the RCS-Cox curves and machine learning interpretation results, we observed that the paradoxical predictive effect of tumor length was primarily concentrated within the 0–3 cm range, while >5 cm remains the commonly used “large tumor” threshold in prior studies and clinical analyses. Therefore, in subsequent survival analyses, this study adopted ≤ 3 cm and > 5 cm as the grouping criteria for small versus large tumors to accentuate the differences between these two biological phenotypes and minimize confounding from intermediate diameter ranges. Kaplan–Meier analysis revealed that within the young, locally advanced tumor population, the small-tumor group exhibited significantly poorer OS than the large tumor group, indicating that small tumor size does not necessarily correspond to favorable long-term outcomes.

These findings collectively indicate that the clinical significance of CRC tumor length requires integrated interpretation with T stage, age, sex, and pathological features. While larger tumors generally reflect higher tumor burden, smaller tumors in specific subgroups may paradoxically associate with increased lymph node metastasis risk and inferior prognosis. Consequently, clinical assessment should not underestimate the risk of small-sized tumors. For locally advanced, young male patients with small tumors accompanied by LVI, PNI, poor differentiation, or special histologic types—even in the absence of detectable lymph node metastasis—we recommend more comprehensive risk evaluation and advocate for more intensive individualized management regarding surgical strategy, adjuvant therapy selection, and surveillance frequency. Although tumor size cannot replace TNM staging, it serves as a crucial complementary indicator to existing risk stratification systems.

This study has several limitations. First, as a retrospective analysis, despite the large sample size and external validation, it remains susceptible to selection bias, information bias, and residual or unmeasured confounding. Tumor diameter was primarily obtained from postoperative pathological records, and variability in specimen handling, measurement procedures, and reporting standards may have introduced measurement error. Second, the predictive performance of the machine-learning models differed substantially across metastatic endpoints. Although relatively good discrimination was achieved for lymph node metastasis, performance for distant metastasis was considerably more limited, suggesting that routinely available clinicopathological variables may not fully capture the biological determinants of systemic metastatic spread. Third, because the models incorporated postoperative pathological variables such as pT stage, LVI, and PNI, they should be interpreted primarily as postoperative risk-stratification models rather than preoperative prediction tools. Fourth, comprehensive molecular characterization remained unavailable, and potentially relevant features such as KRAS/BRAF alterations, CMS classification, tumor immune microenvironment, spatial heterogeneity, radiomic characteristics, and circulating tumor DNA were not systematically evaluated. Therefore, the biological mechanisms underlying the small-volume but potentially aggressive tumor phenotype remain incompletely understood. Finally, the adverse prognostic effect associated with smaller tumor diameter was most evident in locally advanced disease, and its applicability to patients with earlier-stage CRC remains uncertain, which limits the generalizability of this finding to the broader CRC population. Future research should confirm these findings in prospective, multi-center cohorts and integrate molecular pathology, spatial transcriptomics, radiomics or ctDNA analysis (26) to further elucidate the biological basis and clinical translational value of distinct growth patterns in CRC.

## Conclusion

Tumor size may provide complementary information for CRC risk assessment beyond the conventional staging system. Small tumors may exhibit greater lymph node metastatic potential and relatively poorer long-term prognosis in specific patient subgroups, particularly male patients with early-onset, locally advanced disease. In clinical tumor assessment, tumor aggressiveness should not be underestimated solely because of small tumor size. Instead, tumor size should be interpreted in combination with T stage, histological type, differentiation grade, LVI, PNI, and other clinicopathological factors to optimize lymph node metastatic risk assessment, prognostic evaluation, and individualized treatment decision-making for patients with CRC.

## Data Availability

The raw data supporting the conclusions of this article will be made available by the authors, without undue reservation.
