# Supplementary material for: New insights into tumor size in colorectal cancer: smaller size, higher risk?
Source: Front Oncol. 2026 Oct 2;16:1908104. doi: 10.3389/fonc.2026.1908104 (PMC13671944; doi:10.3389/fonc.2026.1908104)
Supplement: Supplementary file 1 [file SupplementaryFile1.docx]

**Figure S1. ROC curves, calibration curves, and decision curve analyses of machine-learning models under different definitions of metastatic outcomes.**

**Figure 2. SHAP feature importance and tumor size effect analyses of machine-learning models under different definitions of metastatic outcomes.**

**Figure S3. SHAP feature importance analyses across the FUSCC and Changhai cohorts.**

| **Metric** | **DecisionTree** | **RandomForest** | **ExtraTrees** | **XGBoost** | **CatBoost** |
| --- | --- | --- | --- | --- | --- |
| AUROC | 0.767 (0.749-0.786) | 0.779 (0.761-0.798) | 0.777 (0.758-0.796) | 0.776 (0.758-0.795) | 0.777 (0.759-0.797) |
| Brier score | 0.195 (0.187-0.203) | 0.195 (0.188-0.201) | 0.197 (0.190-0.203) | 0.194 (0.185-0.202) | 0.194 (0.185-0.202) |
| Accuracy | 0.677 (0.658-0.697) | 0.684 (0.665-0.704) | 0.680 (0.661-0.701) | 0.686 (0.667-0.706) | 0.681 (0.662-0.701) |
| Sensitivity | 0.567 (0.541-0.595) | 0.588 (0.562-0.616) | 0.579 (0.552-0.607) | 0.611 (0.584-0.639) | 0.600 (0.573-0.627) |
| Specificity | 0.834 (0.809-0.858) | 0.819 (0.795-0.844) | 0.825 (0.800-0.850) | 0.794 (0.767-0.819) | 0.796 (0.769-0.822) |
| PPV | 0.830 (0.803-0.854) | 0.823 (0.797-0.847) | 0.825 (0.799-0.850) | 0.809 (0.783-0.833) | 0.808 (0.781-0.832) |
| NPV | 0.575 (0.549-0.600) | 0.582 (0.556-0.609) | 0.579 (0.552-0.605) | 0.588 (0.562-0.615) | 0.582 (0.557-0.609) |
| F1 score | 0.674 (0.651-0.697) | 0.686 (0.664-0.709) | 0.680 (0.658-0.703) | 0.696 (0.675-0.717) | 0.688 (0.667-0.711) |

**Table 1. Performance of machine-learning models (Lymphnode metastasis or Distant metastasis)**

| **Metric** | **DecisionTree** | **RandomForest** | **ExtraTrees** | **XGBoost** | **CatBoost** |
| --- | --- | --- | --- | --- | --- |
| AUROC | 0.787 (0.768-0.805) | 0.799 (0.781-0.817) | 0.796 (0.777-0.814) | 0.797 (0.778-0.815) | 0.800 (0.782-0.818) |
| Brier score | 0.183 (0.175-0.191) | 0.183 (0.177-0.189) | 0.185 (0.179-0.191) | 0.180 (0.172-0.188) | 0.179 (0.170-0.186) |
| Accuracy | 0.729 (0.712-0.747) | 0.731 (0.713-0.748) | 0.728 (0.710-0.746) | 0.728 (0.710-0.746) | 0.732 (0.713-0.750) |
| Sensitivity | 0.626 (0.598-0.653) | 0.628 (0.601-0.656) | 0.627 (0.599-0.654) | 0.642 (0.614-0.669) | 0.644 (0.616-0.671) |
| Specificity | 0.836 (0.813-0.857) | 0.837 (0.814-0.858) | 0.833 (0.811-0.855) | 0.816 (0.794-0.839) | 0.823 (0.800-0.846) |
| PPV | 0.799 (0.772-0.824) | 0.800 (0.773-0.826) | 0.796 (0.769-0.823) | 0.784 (0.756-0.810) | 0.791 (0.764-0.818) |
| NPV | 0.683 (0.659-0.708) | 0.685 (0.661-0.709) | 0.683 (0.659-0.707) | 0.687 (0.663-0.713) | 0.690 (0.665-0.715) |
| F1 score | 0.702 (0.678-0.724) | 0.704 (0.681-0.726) | 0.701 (0.677-0.723) | 0.706 (0.683-0.728) | 0.710 (0.687-0.731) |

**Table 2. Performance of machine-learning models (Lymphnode metastasis)**

| **Metric** | **DecisionTree** | **RandomForest** | **ExtraTrees** | **XGBoost** | **CatBoost** |
| --- | --- | --- | --- | --- | --- |
| AUROC | 0.658 (0.633-0.682) | 0.662 (0.636-0.687) | 0.658 (0.632-0.684) | 0.653 (0.627-0.679) | 0.668 (0.642-0.693) |
| Brier score | 0.230 (0.224-0.235) | 0.229 (0.225-0.233) | 0.231 (0.227-0.235) | 0.226 (0.220-0.232) | 0.226 (0.221-0.231) |
| Accuracy | 0.643 (0.623-0.663) | 0.655 (0.634-0.674) | 0.646 (0.626-0.666) | 0.635 (0.616-0.655) | 0.639 (0.619-0.659) |
| Sensitivity | 0.575 (0.534-0.616) | 0.559 (0.519-0.599) | 0.564 (0.523-0.605) | 0.584 (0.543-0.623) | 0.593 (0.553-0.632) |
| Specificity | 0.666 (0.644-0.690) | 0.689 (0.666-0.711) | 0.675 (0.652-0.697) | 0.653 (0.632-0.676) | 0.655 (0.633-0.677) |
| PPV | 0.378 (0.345-0.411) | 0.388 (0.354-0.421) | 0.379 (0.347-0.413) | 0.372 (0.342-0.404) | 0.377 (0.347-0.409) |
| NPV | 0.817 (0.796-0.838) | 0.816 (0.796-0.836) | 0.815 (0.794-0.836) | 0.817 (0.796-0.837) | 0.820 (0.800-0.841) |
| F1 score | 0.456 (0.422-0.488) | 0.458 (0.424-0.489) | 0.454 (0.421-0.485) | 0.455 (0.423-0.484) | 0.461 (0.429-0.491) |

**Table 3. Performance of machine-learning models (Distant metastasis)**
